# Iron Solubility Measurements in Aqueous MEA for CO_2_ Capture

**DOI:** 10.1021/acs.iecr.4c03980

**Published:** 2025-01-15

**Authors:** Maxime H. J-J. François, Andreas Grimstvedt, Hanna K. Knuutila

**Affiliations:** † Department of Chemical Engineering, Norwegian University of Science and Technology (NTNU), NO-7491 Trondheim, Norway; ‡ SINTEF Industry, NO-7465 Trondheim, Norway

## Abstract

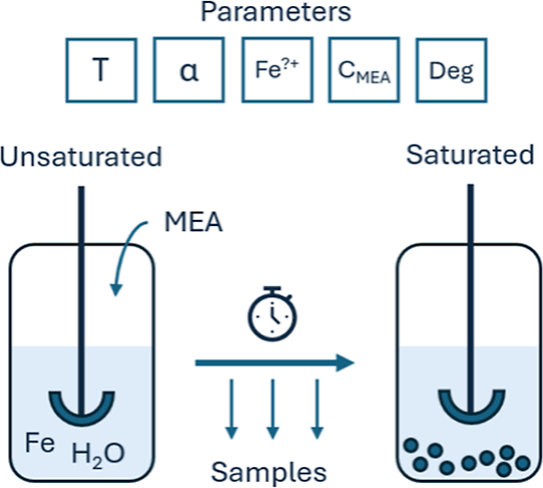

Solvent degradation is one of the major challenges for amine-based carbon capture development. Oxidative degradation appears to be influenced by the combined presence of oxygen and dissolved metals, particularly iron. The maximum amount of dissolved iron corresponds to its solubility in amine solvents. However, the current literature is very limited in providing such data. This work aims to provide a comprehensive method, developed through an investigative process, capable of providing valuable and reliable data on iron solubility in amine solvents. This method is based on precipitation, where an excess concentration of iron salt must precipitate to reach the solubility limit, coupled with analytical methods such as microwave plasma atomic emission spectroscopy and ICP–MS. This work highlights the main limitation of any work on this topic, i.e., the difficulty to determine the oxidation state composition of iron at any time in the solvent. Despite these limitations, the results seem consistent, providing insight into the solubility of iron(II) and iron(III) ions using deduction and highlighting the influence of temperature, CO_2_ loading, and amine concentration. In addition, experiments were conducted using degraded solvent to investigate what to expect in a CO_2_ capture plant. These results open the door for further experiments to improve the general understanding of the catalytic potential of iron and other metals on amine oxidative degradation.

## 1 Introduction

Amine-based CO_2_ capture is a mature technology ready for large-scale deployment, although several challenges remain.^1^ One of these is degradation, which reduces the overall efficiency of the capture process due to chemical decay of the solvent. Degradation reaction mechanisms are complex but appear to be influenced by the presence of metal ions in the solvent, particularly iron.

The presence of metallic cations in the solvent can result from two different origins. The first is the gradual corrosion of the internal walls of the equipment. The main cause of corrosion is the combined presence of dissolved CO_2_ and degradation compounds, more specifically acid species such as carboxylic acids or HSS (Heat Stable Salts).^2,3^ These acid compounds are believed to significantly increase the corrosion of the pilot or plant walls, thereby causing the release of metal ions into the solvent. Corrosion is usually synonymous with higher operating costs due to the need to maintain pumps, heat exchangers, and vessels.^2^ As it is important to prevent corrosion from taking place, inhibitors are commonly used to mitigate it.^4^ However, of all the options available, heavy metal salts of vanadium or copper are often used, thus, voluntarily inducing the presence of more metallic cations in the solvent. The second possible source of metals is the fly ashes that enter the absorber with the flue gas.^5,6^ However, this only applies to capture units coupled with specific industrial facilities, such as coal-fired power plants, that use fuels or raw materials with high levels of metallic impurities.

The catalytic potential of metallic ions has been studied in the past decade as part of the extensive work undertaken to understand degradation mechanisms better. Oxidative degradation of amines appears to be catalyzed by dissolved metals such as iron, copper, or vanadium.^7−9^ According to a previous work,^10^ copper(II) has the greatest effect on the oxidative degradation of MEA, followed by chromium(III) and nickel(II), both of which result from the corrosion of stainless steel. Then come iron(II) ions, whose effect, although less than that of copper(II), is not negligible. Finally, vanadium(V) is the ion that, among those studied, has the least effect on the degradation rate of MEA. Overall, corrosion and degradation are closely related issues, with each potentially feeding the other. While iron is not the most nefarious metallic ion, it is the one that accumulates the most in the solvent in the capture units, hence the focus on it. Pilot plant campaigns have also shown that there is a clear correlation between metal concentrations and ammonia emissions, as ammonia is one of the most abundant compounds resulting from the oxidative degradation of many amines.^11,12^ In the same idea, a previous work^13^ affirms that a strong correlation between iron concentration and degradation is expected for iron concentrations above 5 ppmw, while below that, iron will have only a marginal effect on degradation or emissions.

While it is generally agreed in the literature that the presence of metal ions in the solvent increases its degradation rate, not all pilot campaign results showed similar trends. During a long-term campaign with CESAR-1 solvent (3 M 2-amino-2-methyl-1-propanol +1.5 M piperazine) at the Niederaussem pilot plant using ion exchange to mitigate degradation, a significant removal of iron, nickel, chromium, and zinc from the solvent using an anionic resin was noted, suggesting that these metallic compounds dissolved in the liquid solution were actually in the form of negatively charged complexes.^14^ The removal of these complexes was followed by an increase in the degradation rate of the CESAR-1 solvent used for the campaign against general expectations. It has been suggested that some degradation products may deactivate metals that might otherwise act as catalysts for oxidative degradation by complexing with them as ligands. However, it is unclear whether the properties of the ligands can actually control the potential influence of a complexed metal cation, making some capable of catalyzing oxidative degradation, while others would inhibit the degradation mechanisms. In the meantime, the degradation matrix in the solvent seems to have a larger impact on degradation than the presence of metal ions, according to some observations.^15^

The catalyst mechanism taking place in the solvent is unknown. However, propositions have been made to explain the observations in pilot campaigns. Oxidative degradation refers to the chemical mechanism, where O_2_ initiates the degradation through the formation of radicals. Such reactions usually display a high activation energy but can be catalyzed like many others by metal ions.^16^ Voice and Rochelle^17^ proposed that dissolved metal ions may decompose organic hydroperoxides resulting from the reaction between amine and O_2_, with radicals being formed and the metal ions entering a catalytic cycle, with a succession of oxidation and reduction (quite similar to the Fenton reaction). They also highlighted the participation of metals by showing that metal chelation was more effective than oxygen scavenging to mitigate oxidative degradation. Reynolds and Verheyen^18^ divide the oxidative degradation mechanisms into two categories: oxidation of the amine-metal complexes and oxidation by free radicals. The occurrence of free radicals is often catalyzed by metal ions, as shown by Chi and Rochelle;^9^ therefore, both pathways can be influenced by metal ions. Bedell^19^ also suggested that amines can be directly oxidized by metal ions in the absence of O_2_. The reduced metal ions can then be reoxidized, forming hydroxy compounds, further decomposing.

Regardless of the nature of the reaction mechanism, the catalytic potential of these metallic ions is likely to be correlated with their concentration in the solvent.^12,13^ However, it is also likely that only the metals dissolved in the solvent are capable of catalyzing such reactions. Therefore, this potential should be limited by the solubility of these metals in the solvent, assuming that neither the corrosion rate feeding iron ions in the solvent nor the mass transfer rate related to the oxidative degradation reaction are actually the limiting factors.

To the best of our knowledge, significant precipitation of iron and other metals has never been reported in pilot plants. This may be due to precipitation occurring in cramped spaces in the capture plant and not being observed or addressed. It may also indicate that the solubility threshold, however low, was never reached. Since the concentrations of iron and other metals are of interest for their potential impact on degradation, several pilot campaigns have thoroughly monitored the concentration of iron in the solvent. These values provide a first approximation of the amount of iron that can be dissolved in a degraded amine solvent. The iron content in 30 wt % degraded MEA has been reported by different campaigns and varies widely, from 1 to 3 mg/L,^20^ rising to between 7 and 11 mg/L during a campaign in Denmark,^21^ to higher values between 30 and 60 mg/kg during campaigns at TCM and Niederaussem.^11,16,22^ One paper even reported a final concentration close to 1 g/L of solvent.^20^ This wide range of iron concentrations is most likely due to differences in the corrosion extent. Iron concentration in degraded CESAR-1 was reported with a concentration below 5 mg/kg at Niederaussem.^23^ This result, obtained in the same pilot as the high iron content in degraded MEA, most likely indicates less corrosion but could also be the result of a lower solubility of iron in degraded CESAR-1. These pilots are all different in size and design, such as the presence of additional modules for solvent management or the use of different internals. In addition, the operating conditions and length of the campaigns have varied widely, potentially causing large differences in the extent of corrosion. While this may explain the large variation in iron concentration observed, the differences in the pilot results also highlight the need for a better understanding of iron solubility.

The concentration of metals dissolved in the solvent is a critical parameter; because metallic ions seem to catalyze oxidative degradation, it is necessary to monitor and better predict their concentration in the solvent, as well as their solubility, which corresponds to the maximum extent of this phenomenon. There have been several studies in the past that have attempted to measure iron solubility in amine solvents,^24,25^ but the results are rather scattered. This work aims to establish a comprehensive method for measuring iron solubility and, by extension, metal solubility in amine solvent. This work also aims to investigate different operating conditions’ influence on iron solubility in fresh and degraded amine solvents.

## 2 Methodology

### 2.1 Chemicals

The chemicals used in this work are listed in Table 1. All were used without further purification. Monoethanolamine (MEA) was used to prepare the amine solutions at different concentrations. All solutions were prepared gravimetrically by mixing pure MEA with distilled water. Four different salts containing iron, copper, or nickel were used. Carbon dioxide (CO_2_) was used to preload the solutions when necessary. Nitrogen (N_2_) was used to inert the reactor before the start of each experiment and, when necessary, during the entirety of the experiment. Oxygen (O_2_) was used once to induce accelerated oxidation of the metal ions in the solution.

**Table 1:** Chemicals Used in This Work

name	abbreviation	CAS number	purity	supplier
monoethanolamine	MEA	141–43–5	>99.0%	Sigma-Aldrich
iron(II) sulfate heptahydrate	FeSO_4_, 7H_2_O	7782–63–0	>99.0%	Sigma-Aldrich
iron(III) sulfate hydrate	Fe_2_(SO_4_)_3,_*x*H_2_O	15244–10–7	97%	Sigma-Aldrich
copper(II) sulfate	CuSO_4_	7758–98–7	>99%	Sigma-Aldrich
nickel(II) sulfate hexahydrate	NiSO_4_, 6H_2_O	7786–81–4	>99%	Sigma-Aldrich
carbon dioxide	CO_2_	124–38–9	>99.7%	Linde
nitrogen	N_2_	7727–37–9	>99.7%	Linde
oxygen	O_2_	17778–80–2	>99.7%	Linde

### 2.2 Experimental Work

The solubility of a substance refers to the maximum amount of that substance that can dissolve in a specific amount of solvent to form a homogeneous solution. The solubility of a substance in a given solvent is generally measured as the concentration of the solute in a saturated solution, in which no more solute can be dissolved.

**Figure 1:**
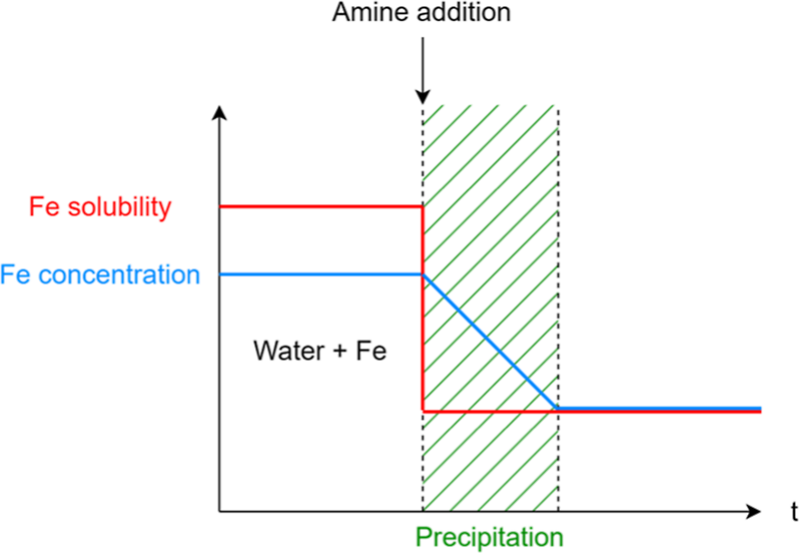
Precipitation-based experimental principle.

Previous solubility measurements were performed by gradually pouring solid iron sulfate into the solvent to be dissolved.^24,25^ Dissolution proved to be quite slow, which increased the duration of the experiment and the likelihood of degradation that could bias the solubility measurements. On the contrary, this work is based on the principle of precipitation. Precipitation occurs when the concentration of a dissolved compound exceeds its solubility; the solvent is supersaturated, and the excess solid separates from the solution to form a precipitate. Thus, in this work, water containing a large amount of dissolved metal was brought into contact with an amine solution. The solubility of iron in an aqueous amine solution is much lower than in pure water.^26^ Therefore, the dissolved iron must precipitate in order to reach the solubility limit in an aqueous amine solution. Figure 1 illustrates this principle.

#### 2.2.1 Apparatus Description and Experimental Protocols

A 1 L jacketed and semiclosed reactor was used to conduct these iron solubility experiments. A water bath was used for temperature control. Stirring of the reactor contents was provided by a Teflon anchor connected to a Teflon shaft driven by a motor located above the reactor. The use of nonmetallic parts was deemed necessary to avoid any interference with the experiments.

As previously mentioned, the solubility experiments performed were based on precipitation. The protocol presented here was applicable to most experiments; others required protocol adjustments due to the nature of the parameter being studied. First, a 50 wt % MEA solution is prepared. Iron sulfate is dissolved in distilled water to make a concentrated iron solution (≈1 g_Fe_/L). Since oxygen can potentially interfere by oxidizing ferrous ions, the reactor is inerted with nitrogen, and then the water bath is brought to the desired temperature. After the stirrer is started (240 rpm), both solutions are added to the reactor to obtain 500 mL of a 30 wt % MEA solution. Excess iron that cannot remain solubilized in the aqueous amine solution will gradually precipitate during the experiment.

Two iron salts were used, depending on the desired initial oxidation state: iron(II) sulfate heptahydrate (FeSO_4_, 7H_2_O) or iron(III) sulfate hydrate (Fe_2_(SO_4_)_3,_*x*H_2_O). For experiments investigating the effect of MEA concentration at 15 and 45 wt %, the concentration of the initial concentrated MEA solution was decreased and increased, respectively, to avoid dilution problems. For experiments investigating the influence of degradation, the used degradation solutions were prepared by mixing in different proportions an original degradation solution corrected with pure MEA to reach a concentration of 37.5 wt %, and a fresh 37.5 wt %% MEA solution. The final addition of the iron solution reduces the total concentration in the reactor to 30 wt %. In addition, due to the expected higher solubility of iron, the concentration of iron in the iron solution was increased up to 4 g_Fe_/L. Finally, for experiments investigating the solubility of copper and nickel, copper(II) sulfate and nickel(II) sulfate hexahydrate were used instead of iron sulfate.

Samples were taken periodically. The sampling was performed using a long plastic pipet to withdraw approximately 10 mL of solution from the reactor. The sample was then filtered by using a syringe with a 0.2 μm filter. The EPA standard method states that anything passing 0.45 μm can be considered solubilized, and preliminary experiments showed no differences between 0.2 and 0.45 μm filters.^27^ The filtered sample was collected in a vial before being stored until analysis. For experiments at elevated temperatures, the pipet and syringe used for sampling were warmed to the experimental temperature to avoid precipitation in them.

The protocol has been modified several times for improvement after the experimental observations. The experiments reported in this work can be grouped into three main phases, with each phase having some slight differences. The results of the third and last protocols are used as the main results of this work, as they are considered to have been carried out under better control. However, there is no reason to distrust the results of the two first protocol.

- Protocol 1: Iron(II) is used for experiments lasting about 48 h. No special consideration is given to the possible interaction of atmospheric oxygen.
- Protocol 2: Iron(II) is used for experiments lasting about 48 h. Special care is taken to mitigate the potential influence of oxygen.
- Protocol 3: Iron **(III)** is used for experiments lasting approximately 72 h. No special consideration is given to the possible interaction of oxygen. The pH is measured for each sample.

Some explanations of these differences are given below. For the purpose of this work, it was initially assumed that after 48 h the concentration of iron in the solvent should have stabilized and precipitation should be complete. This assertion comes from a preliminary experiment conducted with a fresh 30 wt % MEA solution at 25 °C. Samples were taken and analyzed at regular intervals up to 96 h after the start of the experiment. The iron concentration of the samples stabilized after about 36 h. Therefore, it was decided that the experiment should be terminated after 48 h. There was no reason to believe that the time required to stabilize the iron concentration would be different under other circumstances. However, some observations suggested that this might not be the case, and in the third protocol of the experiments, the iron concentration was followed more closely over time, with a total duration of 72 h instead of 48 h.

Another difference revolves around the oxidation state of iron. The first approach was to use a ferrous salt, as is done in the literature. In this case, there is a risk that the oxygen present in the atmosphere and, to a lesser extent, dissolved in the liquid phase, will oxidize iron(II) to iron(III), creating uncertainty in the measurement, especially knowing that differences in solubility between the two forms have been observed before.^28^ Therefore, a protocol modification was developed to reduce this risk. In fact, this is the original reason for inerting the reactor prior to the experiment. Since the reactor is not perfectly sealed, it was decided to blow nitrogen continuously into the reactor above the liquid phase to reduce the likelihood of the oxygen interaction at the liquid–gas interface for experiments studying the influence of the oxidation state of iron. In addition, the water used to prepare the iron and amine solutions was treated to remove dissolved oxygen by stirring, heating, and blowing nitrogen. The third protocol gets around this problem using a ferric salt.

### 2.3 Analytical Methods

Two methods were used to determine the iron content of the samples: ICP–MS (inductively coupled plasma mass spectrometry) and microwave plasma atomic emission spectroscopy (MP-AES). Both methods show consistent results and good reproducibility. Both methods were calibrated with standards for each analytical run. For both techniques used (ICP–MS and MP-AES), the analytical uncertainty is 5%.

For control purposes, the alkalinity was determined by titration with sulfuric acid using a G20 Compact Titrator (Mettler Toledo). Alkalinity can be directly converted to amine concentration when studying nondegraded solutions. Two parallel measurements are made for each sample, and the average is reported. The absolute relative deviation between the two parallel measurements was always less than 3% in this work.

When necessary, LC–MS (liquid chromatography–mass spectroscopy) was used to analyze the amount of degradation products or amine content in degraded solutions since some degradation compounds may be accounted for by the amine titration measuring alkalinity. The uncertainty is 3% for the solvent amine and 5% for the analyzed degradation compounds.

The CO_2_ loading of the samples was determined by using a total organic carbon (TOC-L) analyzer (Shimadzu), which allows the quantification of inorganic carbon (IC) species. The IC content is determined by acidification with H_3_PO_4_, followed by sparging with synthetic air. The IC is completely released as CO_2_ and is then detected by a nondispersive infrared (NDIR) sensor by measuring the amount of IR radiation corresponding to CO_2_. Standard solutions of sodium hydrogen carbonate (NaHCO_3_) were used to calibrate the analyzer. All three methods (amine titration, LC–MS and TOC) have been used previously and described in more details.^29,30^

The pH was measured using a pH Sensor, DGi115-SC, coupled with a G20S Compact Titrator (Metler Toledo) regularly calibrated.

## 3 Results and Discussion

This section presents the results. For each result, the experimental specifications and operational parameters are provided. The results are discussed and compared to the existing literature, and some possible explanations are given.

The same experiment (iron(II), 30 wt % MEA, 25 °C, α = 0) was repeated several times in order to assess the reproducibility of the experimental protocol used in this work. Considering 5 identical experiments, the relative deviation u(x) is 0.1625 mg/L, which is considered satisfactory. Additional data are provided in the Supporting Information, including a brief uncertainty analysis.

### 3.1 Experimental Overview

Table 2 summarizes the experiments performed and the different parameters, such as the type of salt, the initial iron oxidation state, the amine concentration, the temperature, the CO_2_ loading, and other specifications.

**Table 2:** Experiments Description; the Percentage of Degradation Corresponds to the Amount of Original Degraded Solvent in the Solution Used for the Experiment

no	protocol	experiment description
0	1	**iron(II)**, 30 wt % MEA, 25 °C, α = 0
1	3	**iron(II)**, 30 wt % MEA, 25 °C, α = 0, **nitrogen atm**
2		iron(III), 30 wt % MEA, 25 °C, α = 0
3		iron(III), 30 wt % MEA, **60 °C**, α = 0
4		iron(III), 30 wt % MEA, 25 °C, **α = 0.4**
5		iron(III), **15 wt %MEA**, 25 °C, α = 0
6		iron(III), **45 wt %MEA**, 25 °C, α = 0
7		iron(III), 30 wt % MEA, 25 °C, **α = 0.28**
8		iron(III), 30 wt % MEA, **degraded 12.5%**, 25 °C, **α = 0.28**
9		iron(III), 30 wt % MEA, **degraded 25%**, 25 °C, **α = 0.28**
10		iron(III), 30 wt % MEA, **degraded 37.5%**, 25 °C, **α = 0.28**
11		iron(III), 30 wt % MEA, **degraded 50%**, 25 °C, **α = 0.28**
12	1	**iron(II)**, 30 wt % MEA, **40 °C**, α = 0
13		**iron(II)**, 30 wt % MEA, **60 °C**, α = 0
14		**iron(II)**, 30 wt % MEA, 25 °C, **α = 0.2**
15		**iron(II)**, 30 wt % MEA, **40 °C**, **α = 0.2**
16		**iron(II)**, 30 wt % MEA, **55 °C**, **α = 0.2**
17		**iron(II)**, 30 wt % MEA, 25 °C, **α = 0.4**
18		**iron(II)**, 30 wt % MEA, **40 °C**, **α = 0.4**
19		**iron(II)**, 30 wt % MEA, **55 °C**, **α = 0.4**
20		**iron(II)**, **15 wt %MEA**, 25 °C, α = 0
21		**iron(II)**, **45 wt %MEA**, 25 °C, α = 0
22–24		**iron(II)**, **27 wt %MEA, degraded 100%,** 25 °C, **α = 0.4**
25	2	**iron(II)**, 30 wt % MEA, 25 °C, α = 0, **oxygen atm**
26		**copper(II)**, 30 wt % MEA, 25 °C, α = 0
27		**nickel(II)**, 30 wt % MEA, 25 °C, α = 0

### 3.2 Iron Oxidation State

As mentioned previously, one of the main obstacles in studying any issue related to iron for amine-based carbon capture is the lack of analytical methods available to determine its oxidation state in solution. Some techniques exist, but they rely on the use of low pH to ensure the stability of iron ions, which is not the case with an amine-based solvent; it is then impossible to proceed without interfering by acidifying the sample, the effect of which on iron solubility is unknown. The use of colored indicators is another problem when working with colored solutions, which is the case with degraded amine solvents. Finally, other options are too complex or unavailable to be used easily and quickly after sample collection.^31−33^

There are several hypotheses regarding the behavior of iron ions released by corrosion in the capture unit. The presence of dissolved oxygen may allow most ferrous ions to be oxidized into ferric ions. It has also been suggested that iron may also react with both solvent and degradation compound radicals to be either oxidized or reduced, and while some mechanisms have been proposed, there is little experimental evidence to support this hypothesis in either direction. When dissolved in aqueous solutions, ferrous and ferric ions will complex with water molecules to form metal aquo complexes. The resulting charged compounds are colored and could allow for a visual identification of the dominant form of iron in the solution in undegraded, colorless solutions. The hexaaquairon(II) ion [Fe(H_2_O)_6_]^2+^ gives a pale green color while the hexaaquairon(III) ion [Fe(H_2_O)_6_]^3+^ displays a pale purple. However, due to its tendency to be hydrolyzed, [Fe(H_2_O)_5_(OH)]^2+^ is actually the dominant form of ferric ions in solution, giving it its typical brown-orange color.^34^ It also explains the acidic nature of a ferric solution (eq 1).
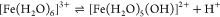
1

However, most of the samples in this work appeared colorless after filtration, which means that the color of the solvent is then more dependent on the suspended precipitated particles. No analytical work has been initiated to determine the nature of the precipitate. However, from a simple assessment of the situation in the reactor, only a few possibilities exist. In the absence of CO_2_, the dissolution of sulfate iron in 30 wt % MEA induces the presence of the following ions: Fe^2+^/Fe^3+^ (depending on the salt used), SO4_2_^–^, H_3_O^+^, and OH^–^ (due to the self-ionization of water, especially at high pH). In pure water, carbonates and hydroxide salts are not really soluble, unlike sulfate salts. It can then be assumed that iron ions, under the form of metal aquo complexes, react with hydroxide ions and precipitate (see eqs 2 and 3 as examples with ferrous ions). Such a reaction would be very favorable in an alkaline medium. The absence of strong coloration of the samples after filtration likely indicates a low concentration, i.e., solubility in these conditions. Both iron hydroxide forms follow the same color pattern as their metal aquo complexes counterparts, still allowing for a visual identification.
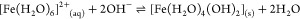
2
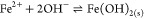
3

**Figure 2:**
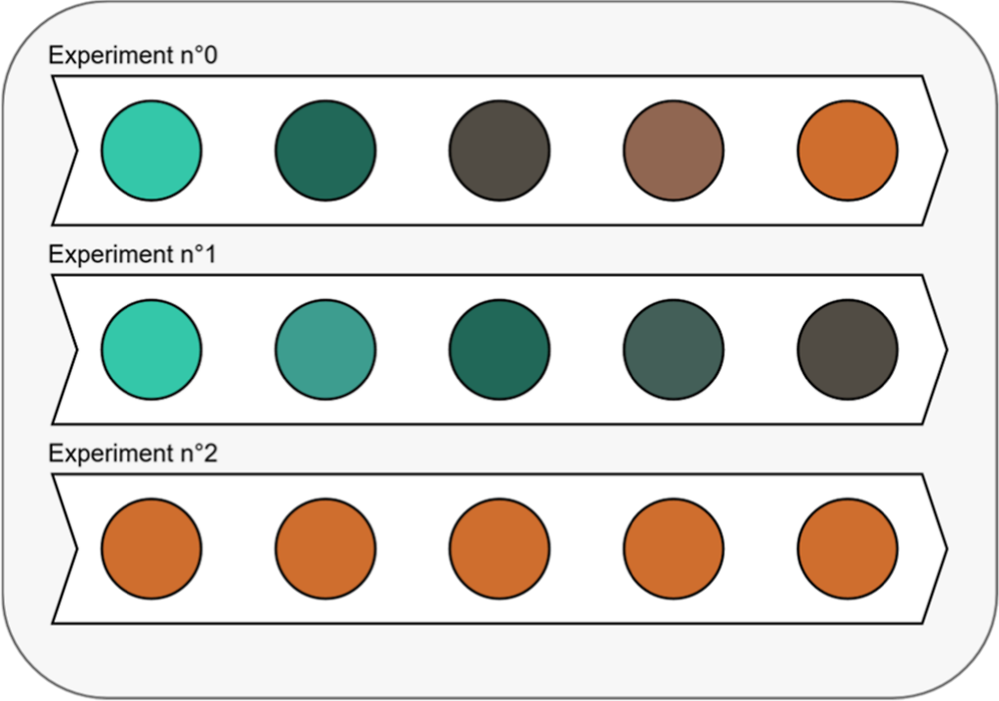
Typical color evolution in the presence of iron(II) (experiments no 0 and 1) and iron(III) (experiment no 2). experiment no 1 was carried out with minimal oxygen interaction.

The effect of oxygen was clearly demonstrated in this work. Figure 2 shows the color evolution of three experiments with 30 wt % MEA; the first and second experiments (experiments no 0 and no 1) were carried out using an aqueous solution of ferrous sulfate, and the third one (experiment no 2) used a solution of ferric sulfate. The two first experiments differ by their conditions. The first experiment was performed according to the original protocol; there is no control attempt on the amount of oxygen available for oxidizing iron(II) in the solvent. The second experiment was carried out with the nitrogen protocol, hence with a limited amount of oxygen available for oxidizing iron(II) ions. For the first experiment, a gradual color change from dark green to rusty red is observed. However, when the presence of oxygen is mitigated like in the second experiment, this color transition is also reduced in its extent, and the solution only turns to dark brown after the same time. Actual pictures can be found in the Supporting Information section (see Figures S2 to S4).

One conclusion can be drawn from this: the presence of oxygen does indeed gradually oxidize iron(II) to iron(III), causing a color change that is clearly visible in colorless fresh MEA. Based on the color change, it can also be assumed that there is still a gradual oxidation of the ferrous ions in the second experiment. However, the ferrous/ferric ratio in this case is higher than that in the first experiment. Therefore, if a difference in solubility is observed between these two experiments, then it is probably due to the difference in the ratio of iron(II) to iron(III) ions. The third experiment is the easiest to observe since the color remains the same throughout the experiment, probably indicating that there are indeed ferric ions in the solution. For the experiment taking place in an undegraded MEA solution, there are no compounds readily available to potentially reduce iron(III) ions.

These three experiments also provide information about the difference in solubility between ferrous and ferric ions. The iron solubility in the second experiment, which is probably the one with the highest amount of iron(II) ions, is stable around 1.5 mg/L after 48 h. The same iron solubility is about 2 mg/L for the experiment following the original protocol. Finally, the third experiment, the one with the highest amount of ferric ions, shows an iron solubility of about 12 mg/L after 72 h. These values seem to be consistent and show a higher solubility of ferric ions than that of ferrous ions. It should be noted that these results are in contrast to what was observed by^24^ in fresh PZ.

It also shows that both experiments carried out with an iron(II) salt give rather similar results, with or without limitation of the amount of oxygen available in the reactor. This could indicate that either the nitrogen protocol does not significantly affect the amount of oxygen available, and the small amount of oxygen remaining is sufficient to oxidize the ferrous ions in the solvent, or the oxidation by oxygen is not significant even without attempting to mitigate it, but this conclusion is at least partially contradicted by the observed color change; the oxidation by oxygen is high enough to change the overall color of the solution but still not high enough to actually convert enough iron ions to reach the same solubility range as with iron(III). One way to quantify this possibility is to estimate the amount of dissolved oxygen in the reactor; using the data from,^35^ one can assume an oxygen concentration of 6 mg/L, which means about 0.1 mmol in the reactor. With about 2 mg/L of dissolved iron, one has 0.018 mmol of iron dissolved in the reactor. These magnitudes clearly indicate that there is enough oxygen to oxidize iron(II), especially considering the continuous replenishment of oxygen in the solvent from the atmosphere without nitrogen sparging. Either way, the other conclusion is that the nitrogen protocol does not significantly change the results obtained with iron II salts.

**Figure 3:**
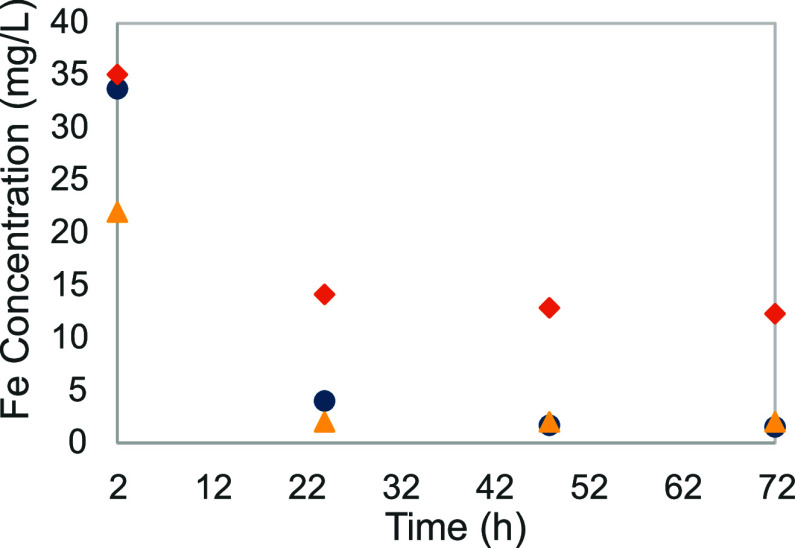
Iron solubility function of time with three different oxidation state makeup: (yellow ▲), iron(II); (blue ●), iron(II) with nitrogen; and (orange ⧫), iron(III).

Figure 3 shows the evolution of the measured iron solubility for the three experiments (experiments no 0–2). As expected, the precipitation mechanism is gradual and not immediate, but the concentration stabilizes after 48–72 h. Not significant differences in behavior are observed.

A fourth experiment (experiment no 25) was conducted using the second protocol of this work with a ferrous sulfate solution. During most of the experiments, pure oxygen was blown into the solvent with the purpose of oxidizing most of the iron(II) ions into iron(III) ions. However, after 48 h, the iron solubility was similar to that obtained with the original protocol, with no significant effect. The presence or absence of oxygen does not seem to change much the result obtained with iron(II), at least in this time frame. It is to be noted that a similar experiment involving the sparging of oxygen in an aqueous solution of iron sulfate show that most ferrous ions were oxidized after only a couple of hours.^36^ This experiment took place under different experimental conditions, and that alone could explain the difference in behavior. In particular, while this experiment took place in water, the viscosity of the MEA could play a role in slowing down the mass transfer of O_2_ to react with iron ions.

### 3.3 Influence of Temperature

One experiment (experiment no 3) took place with a temperature of 60 °C in place of the 25 °C baseline, with multiple samples taken up to 72 h after the solutions contact. As with most of the experiments performed using the third protocol, iron was introduced in the form of ferric ions. One observes a decrease of the iron solubility with increasing temperature. After 48 h, the iron concentration at 60 °C was comprised between 1 and 2 mg/L, while the solubility in the same conditions at 25 °C is 12 mg/L. This influence is rather uncommon for salt, which tends to rather display a higher solubility with increasing temperature, but not unseen. In particular, this trend is also observed in pure water. More importantly, two previous works^25,24^ observed the same effect, only at a different scale (and for CO_2_-loaded solutions).

**Figure 4:**
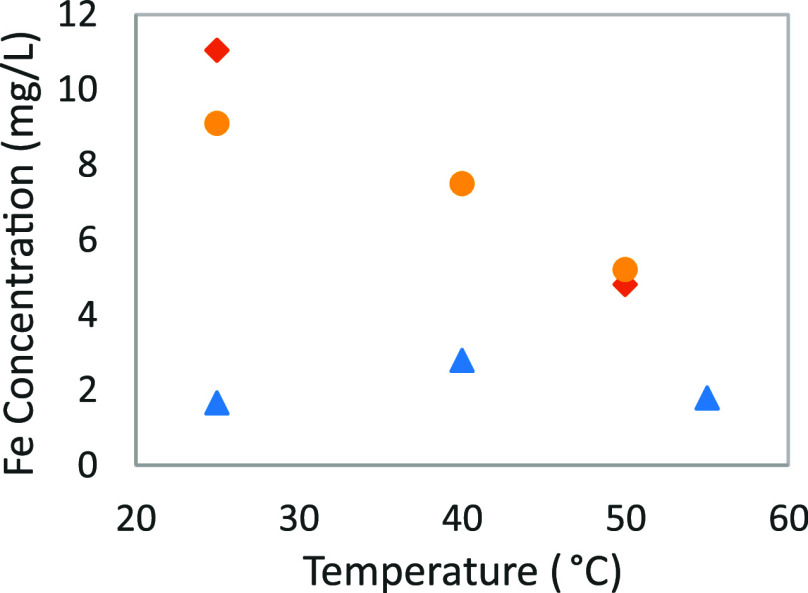
Iron(II) solubility for different temperatures and CO_2_ loadings: (blue ▲), α = 0; (yellow ●), α = 0.2; and (orange ⧫), α = 0.4.

These observations were in line with previous experiments (experiment no 0 and experiments no 12–19) using the first protocol carried out with fresh 30 wt % MEA at different temperatures, but using a ferrous solution. A seen in Figure 4, no significant change can be observed in the absence of CO_2_; the iron solubility remains around 2 mg/L after 48 h. However, in a solution with a CO_2_ loading of 0.2 or 0.4 mol_CO_2__/mol_MEA_, the solubility decreases significantly from 10 to 5 mg/L with increasing temperature. This is a decrease of almost 50% for a temperature increase from 25 to 50 °C. The lack of influence observed with the unloaded solution could be explained by the already low iron concentration, which does not allow a significant variation of the concentration to be noticed. On the contrary, with the loaded solution, where the concentration seems to be higher at 25 °C, there is no problem to observe such an effect.

While not necessarily the full explanation, it is important to remember the temperature dependence of pH, which tends to decrease at higher temperatures (see Figure S1 in the Supporting Information). As seen earlier, a lower pH can be unfavorable to the precipitation of ferrous and ferric ions in the form of iron hydroxide and oxide-hydroxide.

### 3.4 Influence of the Presence of CO_2_

**Figure 5:**
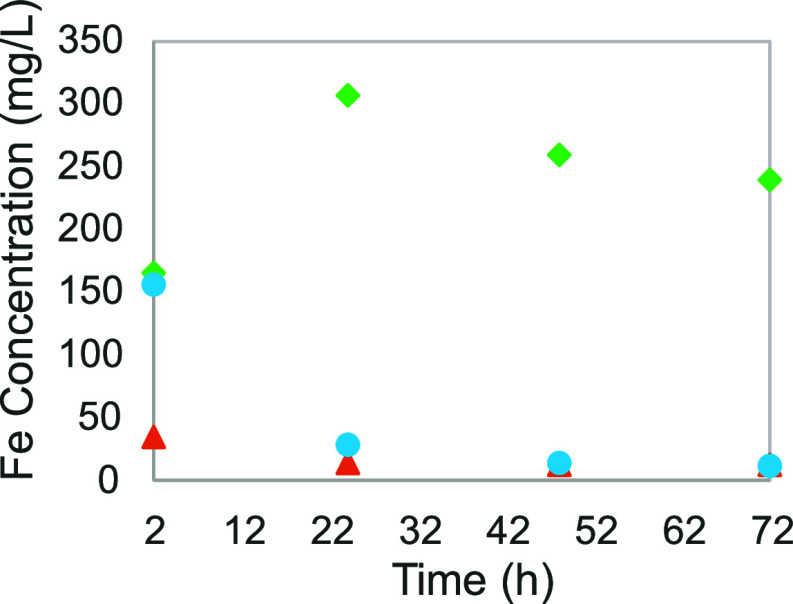
Iron(III) solubility function of time for different CO_2_ loadings: (orange ▲), α = 0; (sky blue ●), α = 0.2; and (green ⧫), α = 0.4.

As already explained in the previous section, the presence of CO_2_ in the solvent seems to significantly increase the iron solubility, from 2 to 10 mg/L at 25 °C, when ferrous ions are at play. In two other experiments (experiments nos 4 and 7) with iron(III) ions, which is already more soluble, the dissolved iron concentration went up from 12 to between 240 and 260 mg/L after 48 to 72 h (see Figure 5). Both sets of experiments indicate that the solubility of dissolved iron is indeed higher in an aqueous amine solution in the presence of CO_2_. A possible explanation is the negative effect on pH induced by the presence of CO_2_, which then renders less favorable the precipitation of iron ions with hydroxide ions. Another possibility is the presence of MEA carbamate, which could potentially form stable complexes with iron ions in the solvent.

It should be noted that the color of the samples after filtration is different from those of the other experiments carried out with undegraded solvent in the absence of CO_2_. In these experiments, the color of the solution in the reactor results from the combined presence of metal aquo complexes and precipitated particles agitated in the reactor by the stirring anchor. After filtration, the sample is largely colorless, probably indicating a low concentration of iron in the solvent available to form these colored complexes. However, iron(III) in the presence of dissolved CO_2_ produces an orange-colored sample after filtration, indicating that an orange-colored complex is likely formed between CO_2_, carbonate or bicarbonate ions, MEA carbamate, and iron cations. This may explain the increased solubility in the presence of CO_2_. The quasi-absence of color in the unloaded solution experiment with iron(III) could also indicate that the precipitated compounds formed in both cases are not the same.

In fact, the nature of the precipitate may also differ due to the presence of dissolved CO_2_ in the solvent. Iron ions in the form of metal aquo complexes may react with either hydroxide or carbonate ions. In the case of the ferrous ions, they can react with both hydroxide and carbonate ions to form Fe(OH)_2_ and FeCO_3_, as shown in eqs 3 and 4. Fytianos^25^ suggested that in the presence of CO_2_, the dominant precipitation product is FeCO_3_ when iron is in the form of ferrous ions in the solvent. It is not clear why Fe(OH)_2_ was not identified. A possible explanation could be the pH which is much lower in the presence of CO_2_, closer to 8 compared to 12 in unloaded 30 wt % MEA, and therefore less favorable to the formation of Fe(OH)_2_.
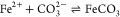
4In any case, the possibilities are quite different in the presence of ferric ions. These are so acidic that they will actually react with the carbonate ions to form CO_2_ (see eq 5). Hence, the carbonate form does not exist, which is also the case for most transition metals with an oxidation state of ^3+^. Fe(OH)_3_ is likely to be the dominant precipitated species. Indeed, Fisher^24^ reported the formation of FeO(OH), a dehydrated form of Fe(OH)_3_, but also of magnetite (Fe_3_O_4_), solid composed of iron(II) and iron(III), which may result from the oxidation of iron hydroxide.
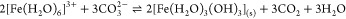
5

### 3.5 Influence of Amine Concentration

The influence of the amine concentration and, more generally, the composition of the solvent is important to study since iron has a solubility in aqueous amine solutions lower than that in pure water, regardless of the conditions. This difference could be explained by the difference in relative permittivity (or dielectric constant) between water and most organic solvents such as amines.^37^ In all systems studied by Bogachev et al., the solubility of several salts is significantly lower than that in pure water. Moreover, the addition of an organic solvent to an aqueous salt solution causes a decrease in the solubility. It is suggested that this is due to the lower permittivity of these organic solvents, which increases the electrostatic ion–ion interactions that should predominantly determine the solubility of the salts in the mixed solvents. Thus, the addition of the organic solvent leads to a decrease in the overall permittivity. Another way of saying this is that amines are less polar than water and are therefore less likely to dissolve well charged species. This mechanism is the principle on which the iron solubility experiments presented in this work are based.

**Figure 6:**
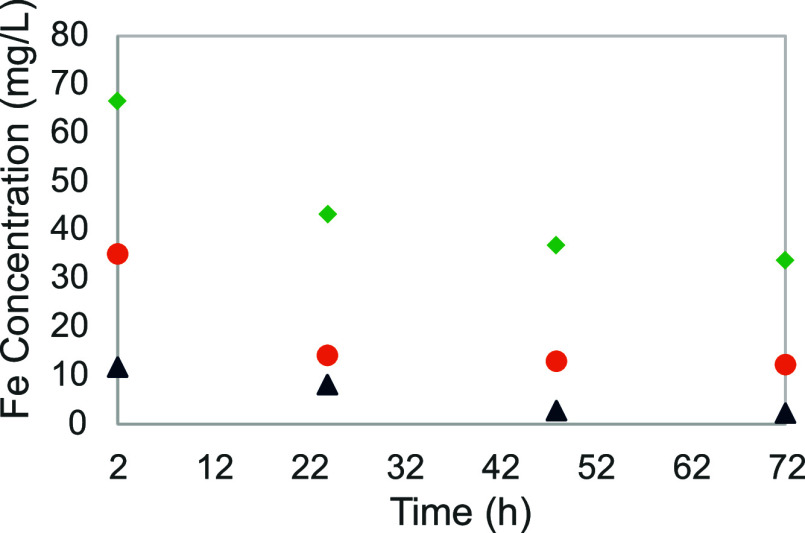
Iron solubility as a function of time for different MEA concentrations: (black ▲), 15 wt %; (orange ●), 30 wt %; and (green ⧫), 45 wt %.

Two experiments (experiments nos 5 and 6) were carried out using the third protocol with iron(III) and aqueous MEA solutions with concentrations of 15 and 45 wt % (see Figure 6). The iron solubility after 72 h was around 2 mg/L for 15 wt % MEA concentration, 12 mg/L for 30 wt % MEA (as previously observed), and 34 mg/L for MEA concentration of 45 wt %. Both increases are consistent, with a 100% increase in amine concentration resulting in a 6-fold increase in solubility, and a 50% increase in amine concentration resulting in a 3-fold increase in solubility. Similar phenomenon has been observed with iron(II). Three experiments (experiments nos 0, 20 and 21) were carried out with MEA concentration of 15, 30, at 45 wt %. The iron solubility was below 1 mg/L with 15 wt % MEA, around 2 mg/L for the baseline concentration, and 20 mg/L with 45 wt % concentration. The progression is not as linear as observed for iron(III), with a solubility multiplied by 10 with an increase of the MEA concentration of 50%, but only an increase by 3 or 4 times with an amine concentration increase of 100%. The absence of correlation in that second case point out an underlying reason for this increase in solubility; for both iron salts, the iron solubility tends to increase significantly with increasing amine concentration.

Considering previous observations, it is unlikely that this is due to the pH change induced by the difference in amine concentration, as seen in Figure S1 in the Supporting Information. In fact, if the difference in amine concentration in each solution causes a slight difference in pH, previous observations would tend to indicate a negative effect of pH on iron solubility, which is contrary to what is observed here. The increase in solubility with increasing amine concentration is rather surprising, considering the high solubility of iron sulfate in pure water. A lower amine concentration means a higher water content; one could then expect a higher iron solubility at a lower amine concentration. Many examples in the literature follow this logic, with an increasing solubility of metal salts in aqueous–organic solvent with higher water content. However, a rather peculiar behavior of metal solubility in organic solvent has also been observed, which remained largely unexplained for now.^37^ Bogachev and Gorbunov^37^ measured the solubility of copper sulfate into aqueous solution of dimethyl sulfoxide (DMSO) with different proportions of DMSO, from 0 to 100%. As expected, a decrease in solubility was observed with the introduction of DMSO and going further from a pure water solution However, at some point with increasing DMSO content, the solubility started to increase again. It is possible that the trend observed for both ferrous and ferric ions between 15 and 45 wt % MEA is caused by the same phenomenon.

### 3.6 Influence of Degradation Compounds

All previous experiments were performed with fresh amine solutions. However, under normal operating conditions, the solvent degrades gradually, and the resulting degradation products begin to accumulate. Therefore, the final parameter studied in this work is the presence of degradation compounds in the solvent (experiments no 7–11). The amine concentration and the CO_2_ loading are kept constant to minimize other influences. For this purpose, a degraded MEA solution from a pilot plant, previously studied by Buvik and Vevelstad,^15^ was used to prepare several amine solutions of the same concentration and loading by mixing it with a freshly loaded amine solution with the same concentration and CO_2_ loading, but in different proportions. Thus, the only varying factor between these solutions is the amount of degradation compounds and, to a lesser extent, the pH, with the alkaline amine acting as a buffer, despite the acidic nature of several degradation products.

**Figure 7:**
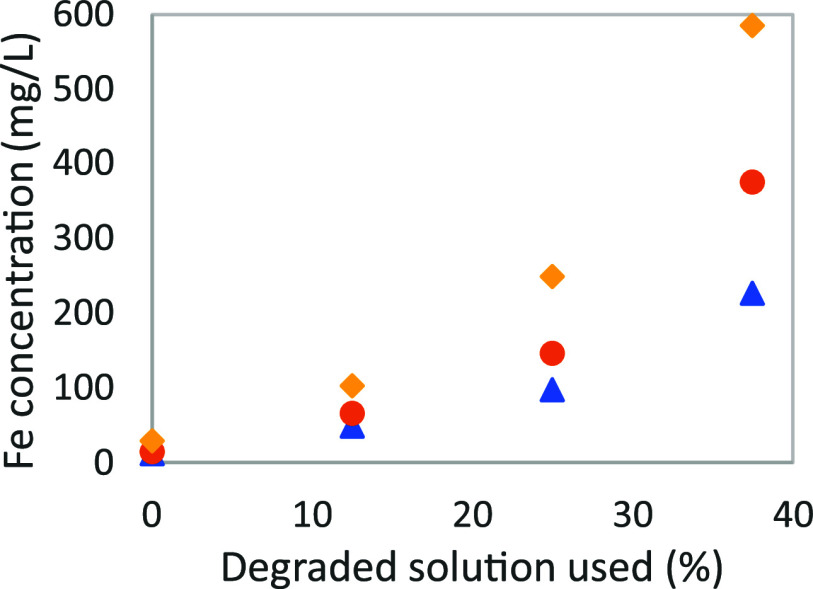
Iron solubility function of the % of degraded solution used for the solvent preparation: (yellow ⧫), after 24 h; (orange ●), after 48 h; and (blue ▲), after 72 h.

The Figure 7 shows the iron concentration after 24, 48 and 72 h for different proportion of degraded solution. The detailed composition of the degraded solution is given in.^15^ The MEA content has been increased up to 30 wt % to facilitate comparison by adding fresh pure MEA. The CO_2_ loading of the degraded solvent and the one of all mixed solution was 0.28 mol_CO_2__/mol_MEA_. The total concentration of quantified degradation compounds in the original degraded solution was approximately 19,000 mg kg^–1^, with HeGly (5835–28–9), HEPO (23936–04–1) and MEA-urea (15438–70–7) being the most abundant. From this follow that the total concentration of quantified degradation compounds was 4750 mg kg^–1^ for the solution prepared with 25% of the original degraded one.

**Figure 8:**
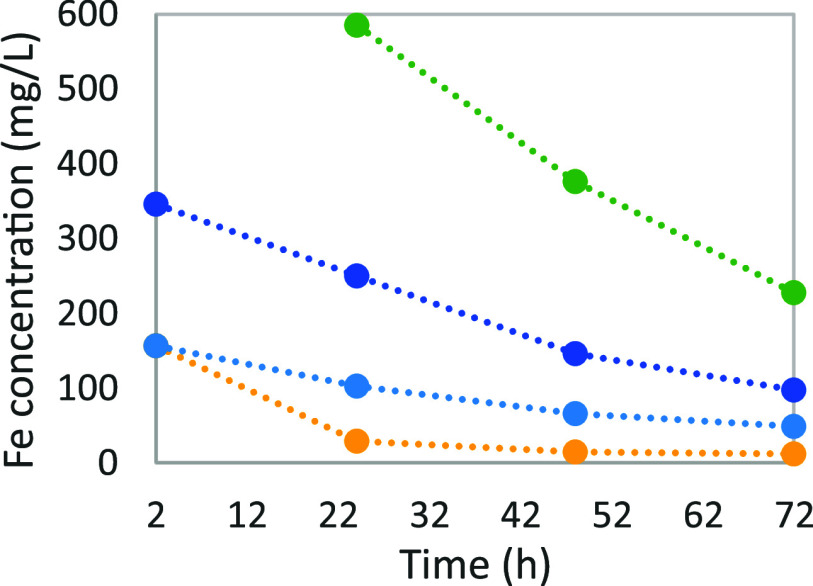
Iron concentration function of time for different amounts of degraded solution: (yellow ●), 0%; (sky blue ●), 12.5%; (blue ●), 25%; and (green ●), 37.5%.

As seen in the figure, the iron solubility for the corresponding fresh solution is relatively low, with around 12 mg/L. The iron solubility after 72 h is then increasing with the amount of degradation compound, with 48 mg/L for 12.5%, 96 mg/L for 25%, and 227 mg/L for 37.5%. The trend seems linear at first, between 0 and 25%, but the pace accelerates at 37.5%, and more important, at 50%, with a measured iron concentration after 72 h above 600 mg/L. That phenomenon can be explained when observing the evolution of the concentration of iron during each experiment (see Figure 8). One can clearly see that the concentration in the solution has not reached equilibrium in most cases and is far from equilibrium in the case of 37.5%. (experiment no 10). When predicting the time required and the concentration at equilibrium using polynomial regression (see Figure S5 in the Supporting Information), one obtains 47 mg/kg for 12.5%, after 80 h, 60 mg/kg for 25% after 110 h, and 111 mg/L for 37.5%, after 120 h. These values, while resulting from a correlation, seem to indicate a rather predictable linear behavior (*r*^2^ > 0.95) between the iron solubility and the amount of degradation compounds in the solvent (Figure S6). This increase in the time required to reach equilibrium can be due to the pH or the presence of degradation compounds. The pH shall influence only the equilibrium point itself, with the concentration in the hydroxide ion impacting the ion product and consequently the supersaturation of the solution. In addition, as shown in Figure S1 in the Supporting Information, the pH of the different solutions is quite similar.

However, the presence of degradation compounds is also associated with an increase in viscosity, which is known to slow the mechanism of many reactions and mass transfer. The same effect can be observed to a lesser extent with the highest CO_2_ loading and amine concentration (Figure 5 and respectively Figure 6), both of which result in increased viscosity.

Several experiments using the first protocol (experiments nos 22–24) carried out with less control of the operating parameters show similar results with ferrous ions, with a solubility between 200 and 300 mg/L in the absence of dilution with fresh amine solvent.

Overall, these results are in line with those in the literature. Both Fytianos^25^ and Fisher^24^ reported higher iron solubility in degraded MEA and PZ, respectively. This is probably due to the ability of some degradation compounds to form stable complexes with iron. Some unpublished in-house experiments seem to indicate a significantly higher iron solubility in the presence of carboxylate in the solvent. Stenså^38^ also showed a higher solubility of iron with the addition of oxalic acid and more generally of carboxylic acids. Bicine, a known amine degradation compound, is also suggested to form complexes with iron.^39^ It is also suggested that these complexes may be negatively charged, which is consistent with observations made by.^14^ Finally, Fisher^24^ suggests the possibility of ferrous iron complexing with EDA, a linear degradation compound of PZ, but also a well-known bidentate chelating ligand for complexes.

### 3.7 Other Metals Solubility

This work focuses on the solubility of iron in aqueous amine solution and highlights some interesting behaviors. For comparison, the solubilities of nickel and copper were also measured in 30 wt % MEA (experiments nos26 and 27). These metals are of interest knowing they can also serve as catalysts of the oxidative degradation of amines. The two first experiments following the same protocol (with the addition of 1 g_meta_l/L water solution) for both copper and nickel show significantly higher metal solubility (around 400 mg/L), not commensurate with the solubility of ferrous iron. Knowing that the concentrated solution of metal represents 40 wt % of the final solution in the reactor, these values indicate that all the copper and nickel were dissolved into the amine solution as seen when comparing the analyzed amounts to calculations assuming that all added copper and nickel would have been dissolved.

It was decided then to perform additional experiments for nickel (assuming both metals were behaving similarly) with even higher initial concentrations in metal, i.e., 10 and 50 mg_Ni_/L. Again, the results seem to indicate that most of the nickel was dissolved in both experiments. These observations could be explained by a potentially higher likelihood of these metals to form complexes, which could then increase their overall solubility in amine solution. These results highlight the peculiar behavior of iron compared to other metals.

## 4 Conclusions

Iron appears to play a key role in solvent degradation mechanisms for amine-based carbon capture. The amount of iron available for such a potential mechanism is then important to quantify. This work aims to provide insight into the behavior of dissolved iron in an aqueous amine solution by providing iron solubility data and investigating the influence of various operating parameters. Several laboratory-scale solubility measurement experiments have been performed with aqueous MEA solutions. This work also discusses the development of a new comprehensive method for iron solubility measurements.

Despite all the uncertainties regarding the oxidation state of iron in solution at any given time, it appears that ferric ions are more soluble than ferrous ions in 30 wt % MEA; this difference remains to be explained, but it was observed in all experiments, even with parameter variations. Additionally, temperature has a negative effect on the solubility of iron, whether in the ferrous or ferric form. This result does agree with the literature. The presence of CO_2_ significantly increases the solubility of iron, especially ferric iron. The MEA carbamate induced by the presence of CO_2_ may form stable complexes with iron, hence increasing its solubility. The lower pH induced by the presence of carbonate ions in the solvent may also explain this as the precipitation of iron hydroxide would be less favorable. Carbonates themselves can form complexes with iron cations and precipitate, but this reaction is impossible with ferric ions. However, since ferrous ions can indeed precipitate when combined with carbonate ions, the solubility limit in that case is higher than in the baseline case but lower than for ferric ions. The amine concentration has partially the same effect; since pH increases with concentration, we can expect a higher solubility at low amine concentration. In addition, the composition of the solvent being closer to pure water at low concentration, one could also expect a higher solubility at a lower concentration. However, it seems that the behavior of the iron solubility function of the amine concentration is not predictable and could follow trends similar to those of other salts in organic solvents. However, this phenomenon is not yet fully understood. Finally, this work highlights the higher solubility of iron in degraded solvent, probably due to the presence of degradation compounds that may form stable complexes, a confirmation made possible thanks to the careful isolation of this parameter by keeping the amine concentration and loading constant. This relationship appears to be linear and clearly indicates that iron solubility is not the limiting factor in pilot plants, as the measured solubility is well above the measured iron concentration observed in several campaigns. Consequently, it is critical to control the iron content in the solvent to mitigate any potential influence on the degradation. Overall, the clear dependence of iron solubility on several parameters such as pH, CO_2_-loading, and temperature also means that iron solubility is expected to vary along the capture cycle.

With respect to the methodology and experimental protocol used and developed in this work, the conclusions are mixed. The method allows for accurate and reproducible results of iron solubility. However, the lack of control over the amount of oxygen available in the reactor, as well as the lack of a method to distinguish between ferrous and ferric ions, remains a challenge. Further, even 72 h are not sufficient for the precipitation to reach equilibrium in the most viscous solutions. This means that the motivation to use precipitation instead of solubilization for faster experiments is not as relevant as expected. Also, the use of the syringe with a filter to filter the samples should be further investigated, as pressure may also affect solubility. On the other hand, an obvious improvement in this precipitation-based method is that it does not necessarily rely on visual observation to confirm that equilibrium has been reached.

## Supporting Information Available

Uncertainties, pH measurements, pictures of the color evolution of several experiments, and graphs of the evolution of iron solubility in the presence of degradation compounds (PDF)
