# Supplementary material for: Iron Solubility Measurements in Aqueous MEA for CO2 Capture
Source: Ind Eng Chem Res. 2025 Jan 15;64(4):2318–28. doi: 10.1021/acs.iecr.4c03980 (PMC11789149; doi:10.1021/acs.iecr.4c03980)
Supplement: Supplementary file 1 — ie4c03980_si_001.pdf [file ie4c03980_si_001.pdf]

Iron solubility measurements in aqueous MEA for CO_2 capture

Maxime H. J-J. François[a], Andreas Grimstvedt[b], Hanna K. Knuutila[a]*

[a]Department of Chemical Engineering, Norwegian University of Science and Technology (NTNU), NO-7491 Trondheim, Norway

[b]SINTEF Industry, NO-7465, Trondheim, Norway

*CORESSPONDING AUTHOR EMAIL ADDRESS:

hanna.knuutila@ntnu.no

Supplementary Information

A. Short uncertainty analysis

The uncertainties associated with the methods used in this paper have already been investigated in a previous paper (François, M. H. J., Patil, D., Brulé, S., & Knuutila, H. K. (2024). CO2 solubility and amine volatility data for low-concentration solutions of MEA, AMP, PZ and CESAR-1 blend (AMP/PZ). *Results in Engineering*, *22*, 102163.). The most important results are presented here.

Uncertainty on the amine concentration:

$$u(C_{amine}) = C_{amine}\sqrt{(\frac{u(C_{titrant})}{C_{titrant}})^2 + (\frac{u(V_{eq})}{V_{eq}})^2 + \left(\frac{u(m_{sample})}{m_{sample}}\right)^2}$$

With

$$u(V_{eq}) = 0.0006ml$$

$$\frac{u(C_{titrant})}{C_{titrant}} = 0.00051$$

$$u(m_{sample}) = 0.0021\ g$$

And where V_{eq} and m_{sample} are specific to each titration used for determining the amine content of a sample.

Uncertainty on the loading of carbon dioxide:

$$u(\alpha) = \alpha\sqrt{(\frac{u(C_{amine})}{C_{amine}})^2 + (\frac{u(C_{CO2})}{C_{CO2}})^2}$$

With

$$\frac{u(C_{CO2})}{C_{CO2}} = 0.0202$$

Uncertainty on the temperature

The uncertainty on the measurement by the thermocouple of the temperature inside the system is 0.2K.

Uncertainty on the iron concentration measurements

For both techniques used (ICP-MS and MP-AES), the analytical uncertainty is 5%.

Reproducibility

The same experiment (iron(II), 30 wt% MEA, 25 °C, $\alpha=0$) was repeated several times in order to assess the reproducibility of the experimental protocol used in this work. Considering 5 identical experiments, the relative deviation u(x) is 0.1625 mg/L, which is considered satisfactory.

B. pH measurements

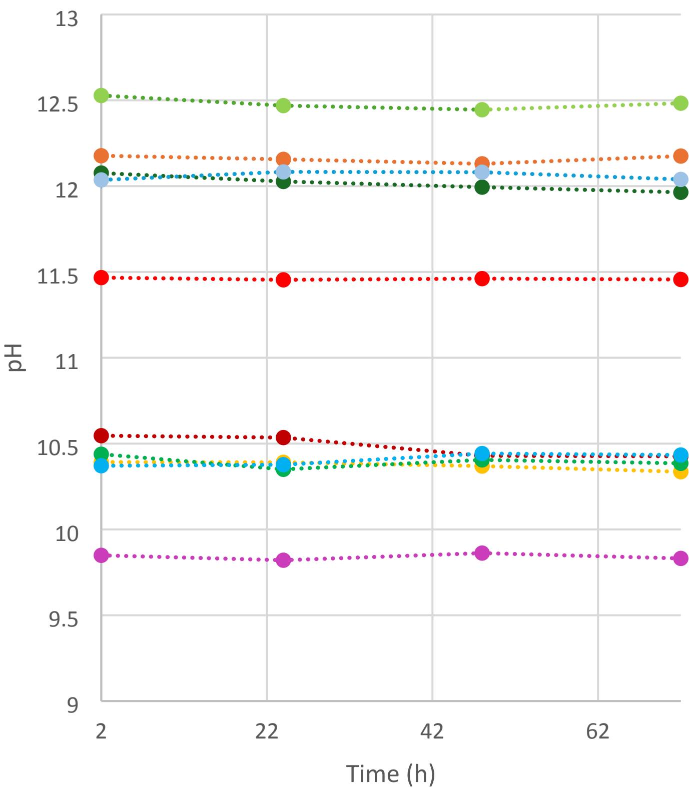

Figure S1: pH evolution during phase 3 experiments: ●, Experiment n°1; ●, Experiment n°2; ●, Experiment n°3; ●, Experiment n°4; ●, Experiment n°5; ●, Experiment n°6; ●, Experiment n°7; ●, Experiment n°8; ●, Experiment n°9; ●, Experiment n°10.

C. Additional pictures

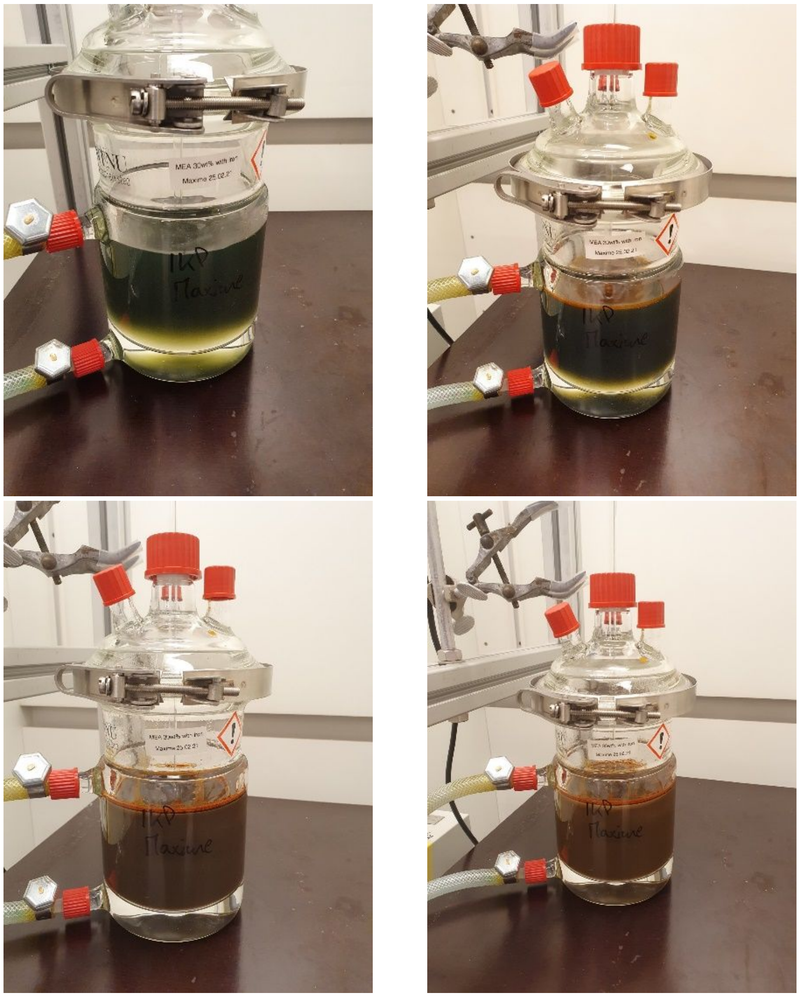

Figure S2: From left to right, first row: iron(II) experiment after 0h, 1h; second row: 24h, 48h.

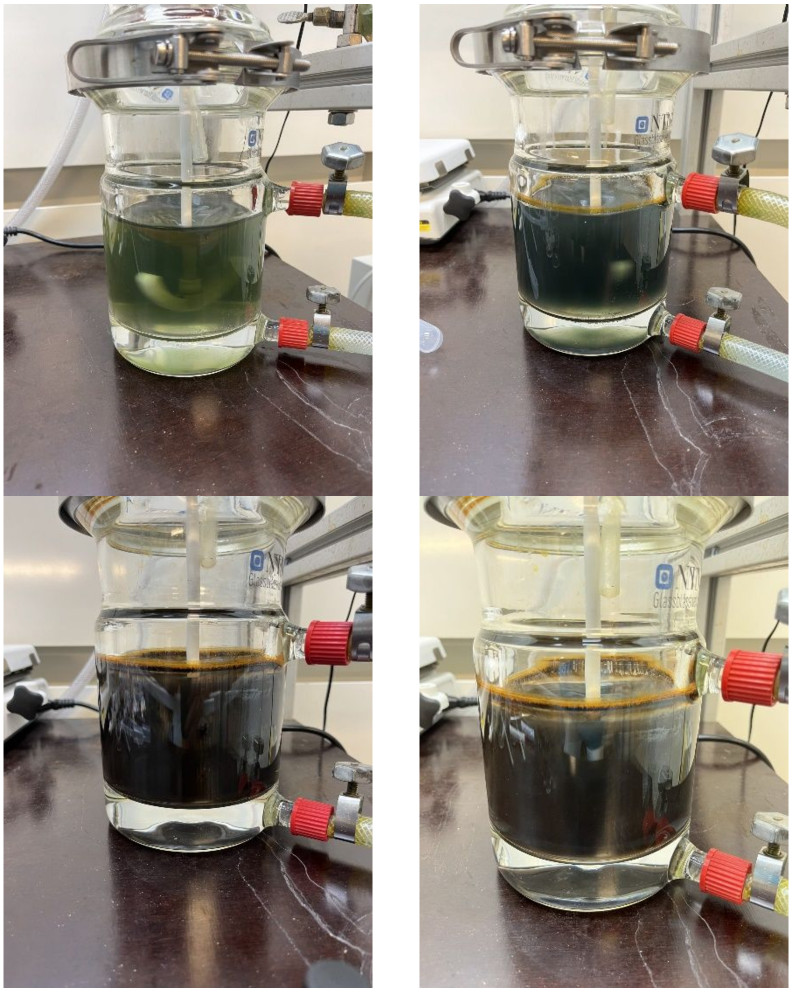

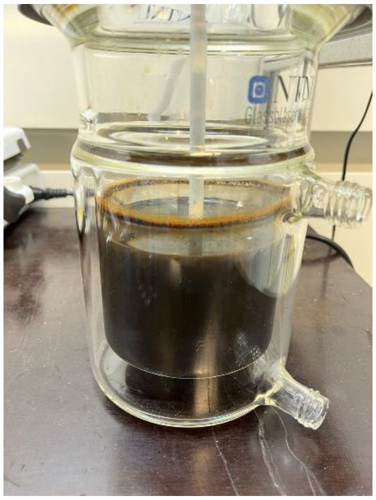

Figure S3: From left to right, first row: iron(II) experiment in presence of nitrogen after 0h, 2h; second row: 24h, 48h; third row: 72h.

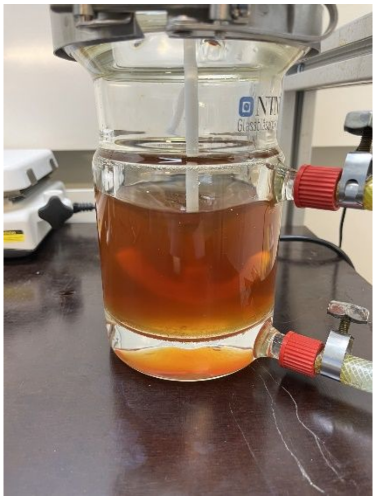

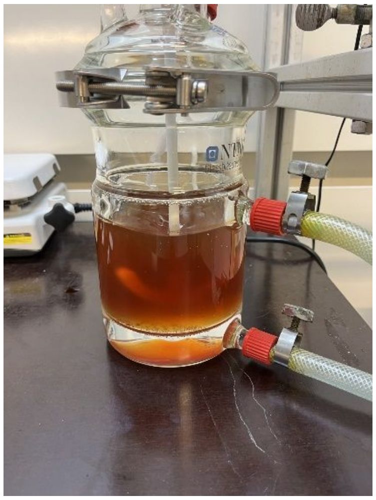

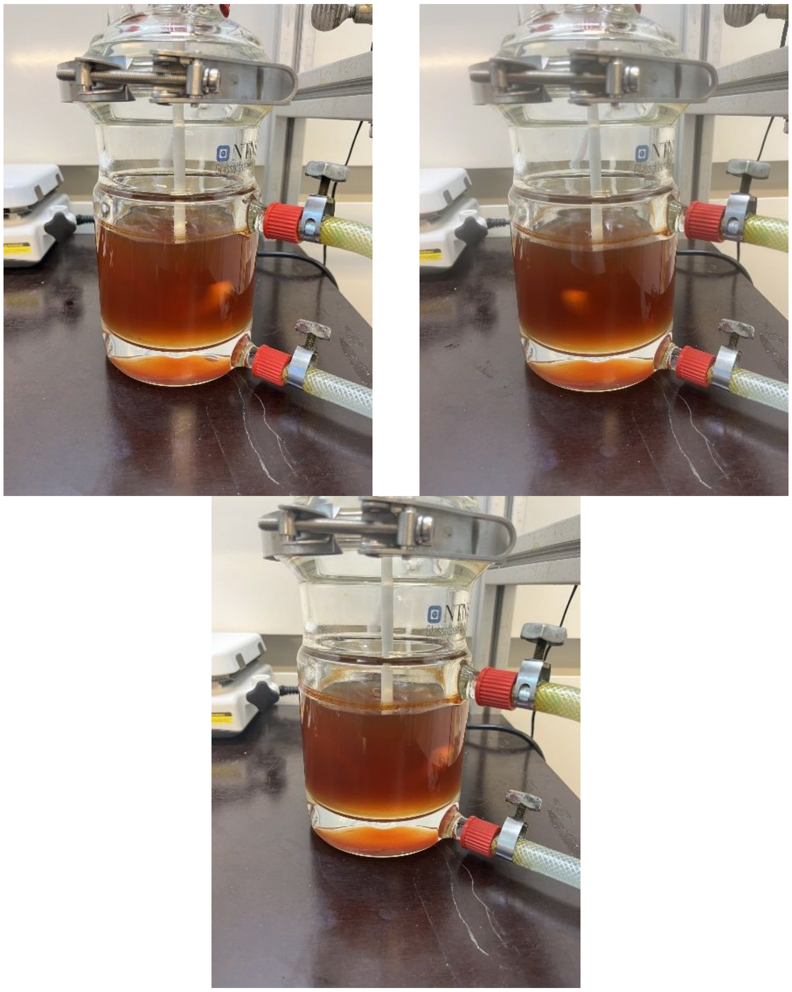

Figure S4: From left to right, first row: iron(III) experiment after 0h, 2h; second row: 24h, 48h; third row: 72h.

D. Influence of degradation compounds - Additional information

The time required to reach equilibrium was estimated on the basis of the observed evolution of the concentration for the three following solutions (12.5, 25 and 37.5%).

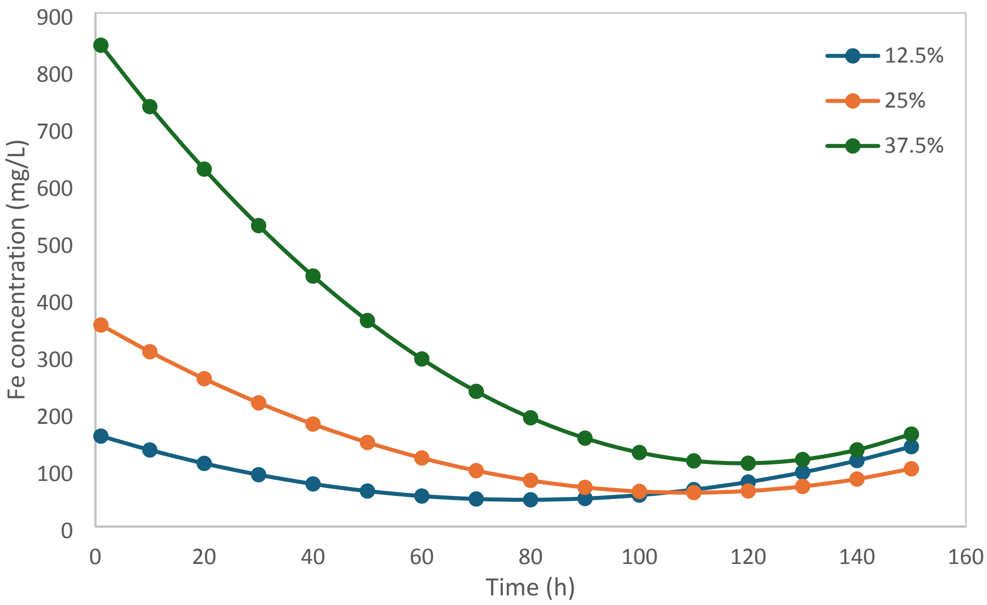

Figure S5: Concentration estimated of dissolved iron function of the time (passed the tipping point, these curves have no physical meaning)

The corresponding iron concentration was then approximated as the concentration that would have been reached if the precipitation process had been able to complete its term. The existing correlation between degradation concentration and iron solubility using these approximations appears to be linear.

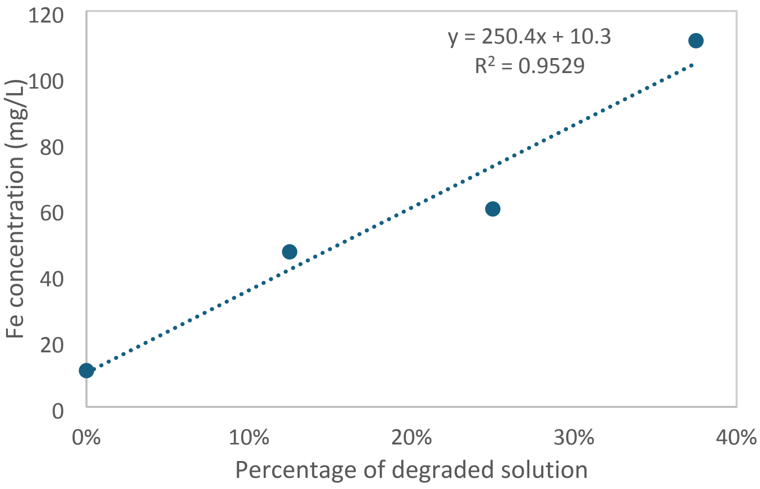

Figure S6: Iron solubility function of the amount of degraded stock used for the preparation of the studied solution (estimation)
